# When cognitive emotion regulation is not enough: a process-oriented perspective on school bullying victimization and suicidal ideation among university students

**DOI:** 10.3389/fpsyg.2026.1778602

**Published:** 2026-03-19

**Authors:** Fudan Wang, Shipei Cui, Nam Jeong Jo

**Affiliations:** School of Education, Woosuk University, Jeonju, Republic of Korea

**Keywords:** cognitive emotion regulation strategies, process-oriented model, school bullying victimization, suicidal ideation, university students

## Abstract

School bullying has been widely recognized as a significant social risk factor for suicidal ideation among university students; however, the psychological processes linking bullying experiences to suicide risk remain insufficiently understood, particularly with respect to the role of cognitive emotion regulation. Using a sample of 600 Korean university students, this study examined the association between school bullying victimization and suicidal ideation and tested whether adaptive and maladaptive cognitive emotion regulation strategies functioned as mediators in this relationship. Data were collected via self-report questionnaires and analyzed using correlation analysis and regression-based mediation analysis. In addition, voluntarily shared narrative accounts were descriptively reviewed to provide contextual interpretation of the quantitative findings. Results indicated that school bullying victimization was positively and robustly associated with suicidal ideation, and this association remained significant after accounting for cognitive emotion regulation strategies. Bullying victimization was significantly related to both adaptive and maladaptive regulation strategies; however, neither strategy demonstrated a significant association with suicidal ideation, and no mediating effects were supported. Interpretive insights from participants’ narratives suggested that, under conditions of chronic and repeated bullying, regulation efforts were often present but progressively undermined, contributing to emotional exhaustion and a narrowing of perceived action possibilities. Together, the findings suggest that the relationship between school bullying victimization and suicidal ideation among Korean university students may be best understood as involving a primarily direct and cumulative process, within which individual-level cognitive emotion regulation strategies may be insufficient on their own to buffer the effects of persistent social adversity. By integrating quantitative analyses with contextualized narrative insights, this study offers a process-oriented lens for understanding bullying-related suicidal ideation.

## 1 Introduction

Suicide cannot be understood solely as an individual act or personal decision; instead, it is deeply embedded in specific social environments and structural conditions. This core perspective was first articulated by David Émile Durkheim in his seminal work Suicide (Spaulding, 2002; Lee and Choi, 2016). Suicide has since been recognized as a significant global public health concern. In many countries, both completed suicides and suicide attempts have shown an increasing or persistently high trend in recent years. More recently, the risk faced by children and university students has drawn particular attention (Gómez-Tabares, 2021). Data from the Centers for Disease Control and Prevention indicate that suicide rates in the United States increased by approximately 36% between 2000 and 2022, affecting individuals across all age groups; notably, suicide has become the second leading cause of death among university students and young adults (Centers for Disease Control and Prevention, 2023). In South Korea, statistics from the Ministry of Health and Welfare reported approximately 14,000 suicide-related deaths in 2023, while survey data indicated that more than 7.6 million individuals had experienced Suicidal ideation or formulated suicide plans (Ministry of Health and Welfare, 2024). Among member countries of the Organization for Economic Co-operation and Development, South Korea has consistently exhibited suicide rates above the average. It is often identified as one of the most affected nations (Park, 2025). Adolescence represents a critical developmental period during which emotional regulation and psychological resources continue to mature. When Suicidal ideation or suicide attempts emerge during this stage, the consequences can be profound at both psychological and behavioral levels. Suicidal ideation rarely occurs in isolation; once present, it may progressively develop into more concrete manifestations such as suicide planning and suicide attempts (Borges et al., 2008). Building on this, for university students navigating the transition to emerging adulthood, peer relationships play a crucial role in socialization. Previous research has shown that, even after controlling for multiple known risk factors, school bullying remains a significant contributor to suicide risk among this population (Kim et al., 2009).

Adolescence is often regarded as a period characterized by vitality and growth; however, it is also a stage in which individuals are frequently exposed to various risk factors. University students spend a substantial portion of their time in school settings, and those who experience school bullying—particularly victims of repeated bullying—often face serious challenges to their psychological well-being. In the present study, school bullying is understood as a form of repetitive peer aggression characterized by power imbalance, consistent with the conceptualizations proposed by Olweus (1993) and subsequent scholars (e.g., Coloroso, 2003). Within the literature on school bullying, victims have commonly been categorized as either passive victims or provocative victims (Olweus, 1994; Salmivalli, 2001). Many passive victims lack effective coping strategies and consequently refrain from protective or resistant responses. Experiences of bullying have been linked not only to emotional difficulties but also to elevated risks of self-harm and suicidal behaviors (Ha, 2014; Eyuboglu et al., 2021). In addition, bullying victims often encounter a range of long-term challenges, including impaired social functioning, peer rejection, and persistent distress related to Suicidal ideation (Klomek et al., 2007; Klomek et al., 2010). For many university students, the psychological impact of bullying is difficult to overcome and may extend into adulthood (Gorman et al., 2021). Bryson et al. (2021) further reported that individuals with a history of bullying victimization exhibited higher levels of self-harm ideation and depressive symptoms compared to those without such experiences. Taken together, School Bullying Victimization, as an adverse life event, has been consistently associated with elevated risks of psychological maladjustment and suicidal ideation among university students (Breitenstein et al., 2025; Huang and Chui, 2024; Wang et al., 2024). However, despite robust evidence linking bullying victimization to adverse mental health outcomes, less is known about the psychological mechanisms through which these experiences translate into suicidal ideation during emerging adulthood. University students are best understood as the outcome of multiple interacting psychological and social processes rather than a single causal factor. Prior studies have identified a range of influences, including life stress, peer relationships, insufficient social support, and psychological states such as depression and hopelessness (Halliday et al., 2021; Ttofi et al., 2011). Subsequent research has further suggested that, within the context of school bullying, the association between bullying experiences and suicidal ideation may not operate solely through a direct pathway but may be transmitted through various psychological processes, such as psychological resilience and problematic internet use (Zhou et al., 2017; Cao et al., 2021). From a cognitive perspective, Vacca et al. (2023) proposed that cognitive emotion regulation strategies (CER), as a central means by which individuals manage stressful life events, may function as a potential mechanism linking School Bullying Victimization to Suicidal ideation.

Emotions and how individuals regulate them play a central role in social adaptation and psychological well-being. Keltner and Kring (1998) noted that positive emotions facilitate the development and maintenance of peer relationships, whereas negative emotions may undermine interpersonal interactions (Elliot, 2006). In the context of bullying, victims who fail to effectively regulate these intense negative emotions may experience heightened psychological distress, further increasing their vulnerability to suicidal ideation. Cognitive emotion regulation refers to the processes through which individuals experience emotions via physiological, behavioral, and cognitive responses, cognitively process emotionally relevant information, and consciously transform or manage these emotions through different regulatory strategies (Mauss et al., 2007). Building on this conceptualization, Garnefski et al. (2001) distinguished cognitive emotion regulation strategies into two broad categories: Adaptive Cognitive Emotion Regulation Strategies (ACER) and Maladaptive Cognitive Emotion Regulation Strategies (MCER). This classification has provided an essential framework for understanding individual differences in emotional regulation when coping with stressful or adverse life events. Cognitive emotion regulation has been widely regarded as playing an important role in individuals’ psychological adjustment. University students who experience stress or adverse events and predominantly rely on Adaptive Cognitive Emotion Regulation Strategies (ACER) tend to maintain more positive psychological states. In contrast, greater reliance on Maladaptive Cognitive Emotion Regulation Strategies (MCER) has been associated with an increased likelihood of adverse psychological outcomes (Sullivan et al., 1995; Kwak and Lim, 2019). Importantly, adaptive and maladaptive cognitive emotion regulation strategies are not necessarily mutually exclusive; individuals may employ multiple strategies simultaneously when coping with stressful or adverse experiences. In general, cognitive emotion regulation strategies contribute to psychological adaptation by supporting emotional stability, with adaptive strategies being associated with positive psychological outcomes and maladaptive strategies being closely linked to negative emotional states (Aldao et al., 2010). Subsequent research has further connected cognitive emotion regulation to suicide risk. Maladaptive cognitive patterns—such as persistent pessimistic thinking and rigid self-critical beliefs—are closely associated with increased suicidal ideation among university students (Beck et al., 1993; Joiner et al., 2009). Such dysfunctional cognitive styles may intensify feelings of entrapment and psychological distress, thereby elevating suicide risk.

In contrast, adaptive cognitive strategies have been proposed as psychological resources that may buffer the impact of stressors on suicidal ideation (Lamontagne et al., 2023). University students who frequently employ strategies such as positive reappraisal, cognitive restructuring, and problem solving tend to report lower levels of suicidal thoughts even when facing stressful circumstances (Garnefski et al., 2001; Daneshvar et al., 2025). Taken together, these findings suggest that suicidal ideation may be shaped by complex patterns of cognitive emotion regulation, involving both the presence of maladaptive cognitive processes and variations in the availability or effective use of adaptive regulatory strategies. However, despite the growing body of research on the association between school bullying and suicidal ideation, studies that systematically examine the mediating role of cognitive emotion regulation in this relationship remain relatively underexplored. Previous research has often focused on specific cognitive regulation strategies in isolation, leaving a limited understanding of how broader patterns of cognitive emotion regulation shape suicide risk among university students (Garnefski et al., 2001). As a salient social stressor, school bullying victimization may influence suicidal ideation through university students’ psychological processing of adverse experiences, while also exerting a direct effect on suicidal ideation (Kelner et al., 2023; Kennedy and Brausch, 2024; Zhao and Ye, 2025). Building on the literature reviewed above, the present study examines the association between school bullying victimization and suicidal ideation among university students and investigates whether cognitive emotion regulation functions as a potential mediator in this relationship. Importantly, beyond testing this mediating pathway, the present study also seeks to further elucidate the experiential and psychological processes through which cognitive emotion regulation may not uniformly operate as a protective mechanism in the context of bullying victimization and suicidal ideation. Accordingly, a conceptual model incorporating both direct and indirect pathways is proposed, and the following research hypotheses are advanced:

*H*1. School Bullying Victimization is positively associated with suicidal ideation among university students.

*H*2. School Bullying Victimization is significantly associated with cognitive emotion regulation.

*H*3. Cognitive emotion regulation is significantly associated with suicidal ideation.

*H*4. Cognitive emotion regulation mediates the association between school bullying victimization and suicidal ideation.

**Figure 1:**
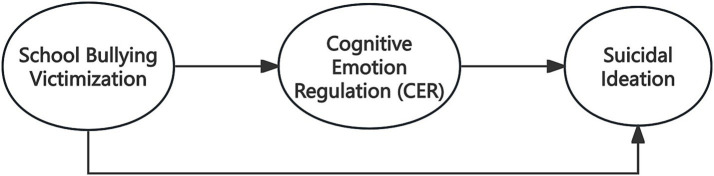
Conceptual mediation model linking school bullying victimization to suicidal ideation via cognitive emotion regulation.

As shown in Figure 1, the proposed conceptual model includes both a direct path from school bullying victimization to suicidal ideation and an indirect path through cognitive emotion regulation. Together, these hypotheses aim to examine both the direct and indirect pathways linking school bullying victimization to suicidal ideation.

## 2 Materials and methods

### 2.1 Participants and procedure

Between September and October 2025, a total of 600 undergraduate students were recruited from three universities in City C, South Korea. Participants were recruited through university counseling and student support services, and the level of school bullying experiences varied among participants. School bullying victimization was assessed using self-report questionnaires and was treated as a continuous variable in the analyses. Before participation, all participants were fully informed about the purpose and procedures of the study, the potential risks and benefits, the principles of anonymity and confidentiality, and their right to withdraw from the study at any time without penalty. Written informed consent was obtained voluntarily from all participants. No personally identifiable information was collected during the research process. All data were used solely for academic research purposes and were handled in accordance with established ethical guidelines and data protection regulations.

After completion of the questionnaire survey, some participants voluntarily engaged in informal conversations with the researcher to share their experiences of school bullying and related psychological feelings. With participants’ consent and full anonymization, these informal narrative exchanges were documented. However, they were not collected through a systematic qualitative research design (e.g., interviews or focus groups) and were not subjected to qualitative data analysis. Accordingly, these materials did not inform hypothesis testing, variable construction, or model estimation, and were used only for illustrative purposes in the discussion to provide contextual understanding of the quantitative findings. The final sample consisted of 600 undergraduate students, including 338 females (56%) and 262 males (43%). Participants were recruited from three universities and represented a range of academic disciplines. Students were drawn from both urban areas (*n* = 398, 66%) and rural areas (*n* = 202, 33%).

### 2.2 Measurements

#### 2.2.1 School bullying victimization

School Bullying Victimization was assessed using a scale derived from the school bullying measurement framework proposed initially by Olweus (1994). The instrument was adapted to the Korean cultural context and subsequently integrated and revised by Kim (2002) based on prior research. In Kim (2002) doctoral dissertation, the scale demonstrated acceptable reliability in a sample of middle school students (Cronbach’s *α* = 0.748). The scale consists of 11 items rated on a 4-point Likert scale ranging from 1 (never) to 4 (five or more times). No items are reverse-coded, and higher scores indicate a greater frequency of bullying victimization. The items cover several common forms of school bullying, including social exclusion and threats or coercion. In the present study, the internal consistency of the scale was excellent, with a Cronbach’s α coefficient of 0.916.

#### 2.2.2 Suicide ideation

Suicidal ideation was assessed using the Scale for Suicide Ideation (SSI) developed by Beck et al. (1979). The scale was later revised and validated in a self-report format by Shin et al. (1990) for use with university students and the general populations in Korea. The instrument consists of 19 items designed to assess the current severity of suicidal ideation. Each item is rated on a three-point scale, with higher scores indicating greater levels of suicidal ideation. No items are reverse-coded. Previous research has reported good internal consistency for the scale (Cronbach’s α = 0.89; Shin et al., 1990). In the present study, the Scale for Suicide Ideation demonstrated excellent reliability, with a Cronbach’s α coefficient of 0.947.

#### 2.2.3 Cognitive emotion regulation questionnaire

Cognitive emotion regulation strategies were assessed using the Cognitive Emotion Regulation Questionnaire (CERQ) developed by Garnefski et al. (2001). The Korean version, revised and validated by Kim (2004) in a master’s thesis, was employed in the present study. The CERQ is designed to assess the cognitive strategies individuals tend to use when coping with adverse or stressful life events. The questionnaire consists of 36 items covering two theoretically distinct but related dimensions: Adaptive Cognitive Emotion Regulation Strategies and Maladaptive Cognitive Emotion Regulation Strategies. All items are rated on a five-point Likert scale ranging from 1 (strongly disagree) to 5 (strongly agree), with higher scores indicating a greater tendency to employ cognitive emotion regulation strategies. No items are reverse-coded. In the present study, although the CERQ comprises adaptive and maladaptive dimensions, cognitive emotion regulation was operationalized as a composite construct to capture individuals’ overall patterns of cognitive emotion regulation in response to stressful experiences. Accordingly, item scores were aggregated to form an overall cognitive emotion regulation index, which was used in subsequent analyses. This approach aligns with the conceptualization of cognitive emotion regulation as a dynamic and integrative psychological process rather than as mutually exclusive regulatory tendencies. Previous research has demonstrated good internal consistency of the CERQ in Korean samples (Kim, 2004). In the current sample, internal consistency reliability was evaluated for the overall CERQ item set (36 items) using Cronbach’s alpha, and the scale demonstrated excellent reliability (*α* = 0.965).

### 2.3 Data analysis

Descriptive statistics were calculated for all study variables and are reported as means and standard deviations (M ± SD). Independent-samples t-tests were conducted to examine group differences across selected demographic variables, with homogeneity of variance assessed using Levene’s test. Pearson correlation analyses were performed to investigate associations among school bullying victimization, cognitive emotion regulation, and suicidal ideation. To test the hypothesized direct and indirect relationships, mediation analysis was conducted using the PROCESS macro for SPSS (Version 4.0; Hayes, 2022), Model 4, with 5,000 bootstrap samples to estimate indirect effects and 95% bootstrap confidence intervals. School bullying victimization was specified as the independent variable (X), suicidal ideation as the outcome variable (Y), and cognitive emotion regulation (CER) as the mediator (M). Indirect effects were considered statistically significant when the 95% bootstrap confidence interval did not include zero. All statistical analyses were conducted using IBM SPSS Statistics 26.0, with a two-tailed significance level set at *p* < 0.05. In the present study, adaptive and maladaptive cognitive emotion regulation strategies were initially examined as conceptually distinct dimensions. However, given the extremely high intercorrelation observed between the two subscales in the current sample, composite scores were computed to represent overall cognitive emotion regulation (CER) and used in the mediation analysis to enhance model stability and reduce multicollinearity concerns. Narrative materials voluntarily shared by a subset of participants were reviewed descriptively only at the interpretation stage to provide contextual illustrations of the quantitative findings; they were not treated as independent qualitative data and were not included in any statistical analyses.

## 3 Results

### 3.1 Demographic characteristics and group differences

To examine potential differences in the study variables across demographic characteristics, independent-samples t-tests were conducted using gender and residential area (urban vs. rural) as grouping variables. School bullying victimization and suicidal ideation were included in the analyses. Cognitive emotion regulation was assessed using the CERQ, and descriptive statistics for its adaptive and maladaptive dimensions across demographic groups are presented in Table 1 for contextual reference. The results indicated that no significant gender differences were found in school bullying victimization or suicidal ideation (all *p* > 0.05). Similarly, no significant differences were observed between urban and rural students with respect to these variables (all *p* > 0.05). Although minor variations in mean scores were observed across demographic groups, the overall pattern suggested substantial similarity in the core study variables across gender and residential background. Overall, these findings indicate adequate sample homogeneity and reduce the likelihood that demographic factors confounded subsequent analyses focusing on the relationships among school bullying victimization, cognitive emotion regulation, and suicidal ideation within the proposed mediation analytical framework. Descriptive statistics are presented in Table 1.

**Table 1:** Demographic characteristics and group comparisons of study variables.

Variables	N (%)	School bullying victimization		Suicide ideation		ACER		MCER	
		t(p)	M ± SD	t(p)	M ± SD	t(p)	M ± SD	t(p)	M ± SD
Gender									
Male	262 (43)	−0.906 (0.365)	2.17 ± 0.75	−0.569 (0.570)	1.66 ± 0.53	−0.086 (0.932)	3.57 ± 0.84	−0.175 (0.861)	3.53 ± 0.85
Female	338 (56)		2.23 ± 0.76		1.68 ± 0.55		3.58 ± 0.84		3.55 ± 0.84
Urban type									
City	398 (66)	1.554 (0.121)	2.24 ± 0.76	1.204 (0.299)	1.69 ± 0.55	−1.139 (0.255)	3.55 ± 0.87	−0.839 (0.402)	3.52 ± 0.87
Rural area	202 (33)		2.14 ± 0.73		1.63 ± 0.51		3.63 ± 0.78		3.58 ± 0.78

ACER and MCER are reported only for descriptive and demographic comparison purposes and were not included as separate variables in subsequent mediation analyses due to their high intercorrelation.

### 3.2 Correlation analysis

Correlation analyses indicated that school bullying victimization was positively associated with suicidal ideation. In addition, school bullying victimization showed significant associations with cognitive emotion regulation strategies as assessed by the CERQ. Adaptive and maladaptive cognitive emotion regulation strategies were strongly positively correlated, suggesting that different regulatory strategies may be concurrently activated when individuals respond to stressful or adverse experiences rather than operating in isolation. This pattern of high intercorrelation supports a methodological treatme**nt** of cognitive emotion regulation as an integrated construct rather than as a set of mutually exclusive strategies. Accordingly, cognitive emotion regulation was treated as a composite construct in subsequent analyses to capture overall patterns of regulatory processing in response to bullying-related stress. Detailed correlation results are presented in Table 2.

**Table 2:** Correlations among study variables (*N* = 600).

Variables	M	SD	1	2	3	4
School bullying victimization (JL)	1.669	0.539	1			
Suicide ideation(ZS)	2.202	0.751	0.924***	1		
ACER (CER)	3.576	0.839	−0.172***	−0.168***	1	
MCER(CER)	3.542	0.842	−0.185***	−0.184***	0.939***	1

Pearson correlation coefficients are reported. ***p < 0.001. School bullying victimization (JL); Suicide ideation (ZS). The strong correlation between ACER and MCER suggests substantial overlap between adaptive and maladaptive cognitive emotion regulation strategies. Accordingly, cognitive emotion regulation was operationalized as a composite construct in subsequent mediation analyses.

Before mediation analysis, multicollinearity diagnostics were conducted for adaptive and maladaptive cognitive emotion regulation strategies. The two subscales exhibited extremely high bivariate correlations, indicating substantial multicollinearity. To ensure the robustness of parameter estimation and avoid instability due to multicollinearity, a composite cognitive emotion regulation (CER) score was computed and used in subsequent analyses. Collinearity diagnostics conducted on the regression model using the composite CER variable indicated no multicollinearity concerns (VIF = 1.00; tolerance = 1.00; condition index < 10).

### 3.3 Mediation analysis of cognitive emotion regulation

To examine the mediating role of cognitive emotion regulation in the association between school bullying victimization and suicidal ideation, a simple mediation analysis was conducted using PROCESS Model 4 with 5,000 bootstrap samples. School bullying victimization was specified as the independent variable, cognitive emotion regulation (CER) as the mediator, and suicidal ideation as the outcome variable. Results indicated that school bullying victimization significantly predicted cognitive emotion regulation. However, cognitive emotion regulation did not significantly predict suicidal ideation when controlling for school bullying victimization. Bootstrap analyses further showed that the indirect effect of school bullying victimization on suicidal ideation through cognitive emotion regulation was not statistically significant, as the 95% confidence interval included zero. In contrast, the direct effect of school bullying victimization on suicidal ideation remained strong and statistically significant.

These findings indicate that cognitive emotion regulation did not emerge as a significant mediator in the relationship between school bullying victimization and suicidal ideation in the present sample (Table 3).

**Table 3:** Bootstrap estimates of direct and indirect effects in the mediation model.

Effect type	Path	β	SE	95% CI	*p*
Direct	JL → ZS	0.6604	0.0114	[0.6380, 0.6828]	< 0.001
Direct	JL → CER	−0.1965	0.0443	[−0.2836, −0.1095]	< 0.001
Direct	CER → ZS	−0.0110	0.0104	[−0.0313, 0.0093]	0.289
Indirect(a × b)	JL → CER → ZS	0.0022	0.0024	[−0.0022, 0.0075]	ns

### 3.4 Interpretive context from participants’ narratives

To provide additional interpretive context for the quantitative findings, participants’ narrative accounts voluntarily shared after questionnaire completion were consulted. These narratives were used to illustrate how university students subjectively experienced and made sense of school bullying and emotional regulation in their daily lives, thereby offering contextual insight into the complexity of the observed statistical relationships. Importantly, these narrative materials were not treated as independent qualitative research data, nor were they used to generate causal explanations or test additional hypotheses. All narratives were collected voluntarily after completion of the questionnaire survey and were reviewed and confirmed with participants before use. Informed consent was obtained for publication, and all identifying information was removed to ensure anonymity. The narratives are presented solely to complement the quantitative findings by illustrating experiential patterns that may not be fully captured by variable-centered statistical models, rather than to establish causal explanations. Narrative materials were reviewed descriptively to identify recurring experiential themes relevant to the quantitative findings, without formal coding or independent qualitative inference.

Several participants described their experiences of school bullying as persistent and pervasive, extending beyond isolated incidents. Bullying was reported to occur repeatedly in the form of humiliation, ridicule, and demeaning remarks targeting various aspects of personal identity, including physical appearance, family background, academic performance, and perceived character. For example, one participant noted:

*It wasn’t just once or twice. Almost every day, someone had something negative to say about me.*

Some students recounted being repeatedly labeled as “ugly” by peers and described ongoing emotional distress associated with such remarks. As one student recalled:

*They kept calling me ugly. I tried to ignore it, but it stayed with me all the time.*

Even when students attempted to reassure themselves that the situation might improve over time, the emotional impact often persisted. One participant reflected:

*I kept telling myself it would get better, but it never really felt that way.*

In participants’ own accounts, repeated exposure to negative peer evaluations was often described alongside enduring feelings of shame, self-doubt, and emotional exhaustion. Some students characterized these experiences not simply as external hostility but in terms of how they reflected on their sense of self-worth, using phrases such as “failure” or “not worthy of respect” when describing their self-perceptions. These self-descriptions were frequently embedded in narratives of daily psychological strain and, in some cases, coincided with the presence of suicidal thoughts. Additionally, several narratives emphasized that bullying episodes were perceived as difficult to understand or resolve, particularly when they involved rumors, stigmatization, or moral accusations (e.g., allegations of cheating or theft) that students felt unable to refute. As one participant described:

*I didn’t even know what I was supposed to explain. People just believed what they heard.*

Participants described these experiences as undermining their sense of control and diminishing confidence in their ability to alter the situation through explanation or personal effort. One student reflected:

*I tried to think differently, but it didn’t really change how bad it felt.*

Such accounts highlight the subjective complexity of bullying experiences and suggest that, from the participants’ perspectives, cognitive coping efforts were often experienced as insufficient in alleviating distress.

When describing how they attempted to cope with bullying experiences, many students reported engaging in various efforts, such as studying harder, trying to think more positively, comforting themselves, or seeking support from counseling services. However, several participants indicated that these efforts were experienced as cognitive futility, particularly when bullying persisted or when support from the surrounding environment was perceived as insufficient. Some students described interpreting repeated difficulties in coping as discouraging, especially in situations where their experiences were denied or where limited support was received from teachers or peers. These accounts reflected a perceived weakening of confidence in cognitive or emotional coping strategies. In addition, narratives referred to a range of emotional and psychological responses, including intense distress, emotional numbness, intrusive memories, nightmares, and physical reactions. Such experiences suggest that the impact of bullying was often perceived as extending beyond immediate incidents. Participants also described feelings of reduced optimism about the future and diminished hope in the context of ongoing distress. In some narratives, suicidal thoughts were mentioned as part of these experiences, described not as impulsive reactions but as expressions of exhaustion or a perceived lack of alternatives. These accounts are presented to illustrate the diversity and complexity of students’ subjective experiences, rather than to propose a specific causal or developmental process.

These accounts highlight the complexity and variability of students’ subjective experiences when coping with prolonged and repeated bullying in contexts where adequate support is perceived as lacking. While many students reported continued efforts to regulate their emotions and thoughts, the narratives underscore that such efforts were not always experienced as sufficient or effective. Rather than offering a causal explanation, these accounts serve to contextualize the quantitative findings by illustrating experiential dimensions of bullying that may not be fully captured by variable-centered mediation models. These considerations are further discussed as limitations and implications in the Discussion section.

## 4 Discussion

### 4.1 Summary of main findings

Of the proposed hypotheses, the hypothesized mediating role of cognitive emotion regulation was not supported, whereas the direct association between bullying victimization and suicidal ideation was consistently confirmed. The results demonstrated a robust and statistically significant direct association between school bullying victimization and suicidal ideation. Although school bullying victimization was significantly associated with overall cognitive emotion regulation, cognitive emotion regulation did not significantly predict suicidal ideation and therefore did not function as a mediator in the proposed model. These findings indicate that, within the scope of the present cross-sectional analysis, cognitive emotion regulation did not account for the association between bullying victimization and suicidal ideation. In the following sections, these results are discussed in relation to existing empirical evidence, relevant theoretical perspectives, and the methodological boundaries of the current study.

### 4.2 Interpreting the non-significant mediating role of cognitive emotion regulation

#### 4.2.1 Rationale for treating cognitive emotion regulation as a composite construct

Although prior research has emphasized the functional distinction between Adaptive Cognitive Emotion Regulation Strategies (ACER) and Maladaptive Cognitive Emotion Regulation Strategies (MCER; Garnefski et al., 2001), preliminary analyses in the present study revealed a very strong positive association between these two dimensions among university students who experienced school bullying victimization. Such a pattern suggests substantial overlap in how different cognitive regulation strategies are mobilized in response to chronic interpersonal stress. To ensure the robustness of the statistical models and to avoid potential estimation bias caused by multicollinearity, cognitive emotion regulation was operationalized as a composite construct in the mediation analysis. This decision was guided not only by statistical considerations but also by a theoretical perspective that views cognitive emotion regulation as a dynamic and integrated psychological processing system. In contexts of prolonged and severe stress, such as repeated school bullying, individuals may simultaneously engage in multiple cognitive strategies—both adaptive and maladaptive—rather than relying on clearly separable modes of regulation. Accordingly, the composite CER score was used to capture participants’ overall tendency to engage in cognitive emotion regulation when coping with adverse experiences. This approach does not deny the conceptual distinction between adaptive and maladaptive strategies but rather reflects the empirical reality that these strategies may co-occur and interact under conditions of sustained victimization.

#### 4.2.2 Direct effects of school bullying victimization and the limits of mediation

Importantly, the non-significant mediating role of cognitive emotion regulation should be interpreted in light of the exceptionally strong direct effect of school bullying victimization on suicidal ideation observed in the present study (*β* = 0.6604, *p* < 0.001). This magnitude suggests that the psychological impact of bullying victimization may be sufficiently intense to dominate the explanatory pathway, thereby constraining the extent to which intermediate regulatory processes can account for additional variance in suicidal ideation. The mediation analysis indicated that school bullying victimization exerted a robust and statistically significant direct effect on suicidal ideation. In contrast, although bullying victimization significantly predicted overall cognitive emotion regulation, CER did not significantly predict suicidal ideation when controlling for bullying victimization, and the indirect effect through CER was not statistically significant, as indicated by bootstrap confidence intervals that included zero. This pattern suggests that, in the present sample, the association between school bullying victimization and suicidal ideation was largely driven by a direct pathway rather than being transmitted through cognitive emotion regulation. The magnitude of the direct effect implies that the psychological consequences of sustained bullying experiences may be sufficiently severe to overshadow the potential buffering role of internal cognitive regulatory processes. For university students exposed to repeated humiliation, social exclusion, or moral accusations, the impact of bullying may manifest in suicidal ideation in a relatively immediate and unmediated manner.

Importantly, the absence of a significant mediating effect should not be interpreted as evidence that cognitive emotion regulation is irrelevant. Rather, it indicates that changes in cognitive regulation alone may be insufficient to account for the strength of the bullying–suicidal ideation association under conditions of chronic and persistent interpersonal stress.

#### 4.2.3 Cognitive emotion regulation under chronic and overwhelming stress

From a theoretical perspective, cognitive emotion regulation is generally understood as a psychological resource that supports emotional adaptation in the face of stress (Aldao et al., 2010). However, when stressors are chronic, uncontrollable, and identity-threatening—as is often the case in prolonged school bullying—these regulatory resources may become progressively depleted. The strong direct association observed in the present study suggests that, for some students, bullying experiences may exceed the functional capacity of cognitive regulation strategies. When negative peer evaluations are persistent and socially reinforced, efforts such as positive reappraisal, self-comfort, or rationalization may no longer alleviate distress effectively. Under such circumstances, cognitive regulation may lose its protective potential, not because it is inherently ineffective, but because the intensity and duration of the stressor surpass the individual’s regulatory threshold. In addition, the use of a composite CER score may have contributed to a statistical cancelation effect, whereby the protective influence of adaptive strategies and the risk-enhancing influence of maladaptive strategies offset one another. This possibility highlights the importance of future research examining specific regulation strategies or profiles in larger samples where multicollinearity can be more effectively addressed.

#### 4.2.4 Integrating quantitative findings with experiential context

The narrative accounts presented in this study offer contextual insight into why cognitive emotion regulation did not emerge as a significant mediator. Participants frequently described continued efforts to cope cognitively—such as trying to think positively, suppressing distress, or seeking meaning in adversity—yet reported that these efforts felt ineffective when bullying persisted, and external support was lacking. These accounts are not presented as causal evidence, nor do they constitute an independent qualitative analysis. Rather, they serve to illustrate experiential dimensions of bullying that may not be fully captured by variable-centered mediation models. In particular, they underscore how repeated failures of coping can undermine confidence in one’s ability to regulate emotions cognitively, potentially contributing to emotional exhaustion, numbness, and suicidal ideation. Taken together, the findings suggest that cognitive emotion regulation may play a more limited role in buffering the effects of school bullying on suicidal ideation in contexts characterized by prolonged victimization and insufficient social support. This interpretation aligns with the cross-sectional nature of the data and highlights the need for longitudinal and person-centered approaches to better capture dynamic regulatory processes over time.

### 4.3 Theoretical implications

Existing theories generally posit that adaptive cognitive emotion regulation strategies (e.g., positive reappraisal, problem solving, and cognitive restructuring) buffer the negative psychological impact of stress, whereas maladaptive strategies (e.g., rumination and catastrophizing) are theoretically associated with greater psychological distress and suicide risk (Garnefski et al., 2001; Aldao et al., 2010; Beck et al., 1993; Joiner et al., 2009). However, the present findings suggest that cognitive emotion regulation may operate as a more complex and integrated process in contexts of chronic bullying victimization. Consistent with these assumptions, the present findings indicate that school bullying victimization is associated with alterations in cognitive emotion regulation patterns, suggesting that prolonged negative social experiences may undermine constructive cognitive coping processes. Although adaptive and maladaptive cognitive emotion regulation strategies were analytically combined into a composite CER score in the mediation analysis due to their extremely high intercorrelation, they are discussed separately here at the theoretical level to remain consistent with established conceptual distinctions in the literature. This distinction is maintained to clarify how prolonged bullying victimization may simultaneously weaken adaptive strategies while reinforcing maladaptive ones, even though these processes were empirically inseparable in the statistical model. Accordingly, the composite CER score should be understood as reflecting an integrated regulatory process rather than the absence of theoretical differentiation. More importantly, however, the study highlights an important boundary condition of cognitive emotion regulation theory by demonstrating that cognitive emotion regulation strategies did not function as significant mediators in the context of chronic and repeated school bullying. This finding does not challenge the validity of cognitive emotion regulation theory per se but rather suggests that its buffering role may be constrained under conditions of sustained and highly personalized social degradation. In such contexts, cognitive emotion regulation may not operate as a stable or sufficient psychological resource. Drawing on the quantitative results and interpretive insights derived from participants’ narrative accounts, the present findings tentatively invite a more process-sensitive understanding of how cognitive emotion regulation may function under conditions of chronic victimization. Although many students reported actively attempting to regulate their emotions, ongoing bullying and limited social support were often described as rendering these efforts ineffective, gradually undermining their sense of agency, self-concept, and perceived meaning. From this interpretive standpoint, suicidal ideation may be understood not simply as the result of deficient regulation strategies but as reflecting cumulative psychological strain and the progressive depletion of regulatory resources. Importantly, this interpretation is offered as a contextual lens rather than as a causal explanation and underscores the need for longitudinal and process-oriented research designs to more directly examine these dynamics over time.

### 4.4 Practical implications

From a practical perspective, the present findings carry important implications for intervention and prevention efforts. Given that cognitive emotion regulation did not function as a significant mediator, interventions that focus predominantly on teaching victims to reframe thoughts or regulate emotions cognitively may be insufficient in contexts of ongoing school bullying. While cognitive-based interventions can be beneficial, the results suggest that they cannot compensate for the psychological impact of persistent victimization when the stressor itself remains unaddressed. Accordingly, prevention and intervention strategies should prioritize environmental and structural approaches that directly target bullying behaviors, such as zero-tolerance school policies, systematic monitoring, and timely institutional responses. Reducing exposure to the core stressor may be a prerequisite for cognitive and emotional coping strategies to regain their effectiveness. In this sense, interventions that aim to enhance cognitive emotion regulation may be more effective when implemented after bullying exposure has been reduced or contained. Furthermore, for the university population specifically, institutions should move beyond generic anti-bullying statements toward establishing low-barrier and anonymous reporting systems, as well as more proactive forms of crisis monitoring. Intervention efforts may also benefit from shifting away from a purely reactive “victim-support” model toward a more systemic approach that addresses the broader social ecology of the campus. From this perspective, cognitive-based strategies may be more effective when introduced after exposure to bullying has been reduced or contained, serving as complementary tools to support the restoration of agency and self-concept, which were described as being progressively undermined under conditions of sustained social adversity.

## 5 Conclusion

The present study examined the associations between school bullying victimization, cognitive emotion regulation, and suicidal ideation among university students. The findings revealed a robust and direct association between bullying victimization and suicidal ideation. Although bullying victimization was significantly related to patterns of cognitive emotion regulation, cognitive emotion regulation—operationalized as a composite CER construct—did not function as a significant mediator in this relationship. These results indicate that changes in cognitive emotion regulation alone were insufficient to account for the elevated risk of suicidal ideation among students who experienced school bullying. Importantly, this pattern suggests that suicidal ideation cannot be understood simply as the outcome of ineffective or maladaptive cognitive regulation. Rather, the findings point to the possibility that, under conditions of chronic and repeated bullying, the psychological impact of victimization may be sufficiently severe to limit the buffering capacity of internal regulatory processes. By integrating quantitative findings with participants’ narrative accounts, the present study offers contextual insight into how students experience and interpret bullying-related distress. These narratives illustrate that regulation efforts are often present but may be perceived as increasingly ineffective when victimization persists, and external support is lacking. This interpretive perspective is offered to contextualize the statistical findings rather than to propose a causal or developmental process model. Taken together, the study highlights the importance of considering the limits of individual-level cognitive regulation in contexts of sustained interpersonal stress. It underscores the need for future longitudinal and multi-level research to more directly examine how regulatory processes, environmental factors, and psychological outcomes unfold over time.

## 6 Limitations

These findings underscore the importance of situating individual psychological processes within their broader social and temporal contexts. Interventions that focus solely on strengthening individual emotion regulation skills may be insufficient when bullying is persistent and social support is lacking. Instead, comprehensive prevention efforts should address both individual coping capacities and the structural conditions that sustain bullying experiences. Several limitations of the present study should be acknowledged. First, the cross-sectional design precludes causal inference, and future longitudinal research is needed to more directly examine the temporal dynamics of bullying-related distress and regulatory processes. In addition, given the high correlation between bullying victimization and suicidal ideation, common method variance cannot be fully ruled out. Further studies employing mixed-methods or person-centered designs may deepen understanding of how cognitive emotion regulation efforts are experienced and potentially undermined under conditions of chronic social adversity. Age information was not collected in the present study. All participants were undergraduate students enrolled in full-time programs, which implies a relatively narrow age range. However, future studies should include age as a covariate to examine developmental differences further. Despite these limitations, the present study contributes to a more nuanced understanding of the conditions under which cognitive emotion regulation may have limited buffering capacity in the context of sustained school bullying, highlighting the importance of integrating individual-level and contextual perspectives in future research and intervention efforts.

## Data Availability

The raw data supporting the conclusions of this article will be made available by the authors, without undue reservation.
